# Towards new cholera prophylactics and treatment: Crystal structures of bacterial enterotoxins in complex with GM1 mimics

**DOI:** 10.1038/s41598-017-02179-0

**Published:** 2017-05-24

**Authors:** Julie Elisabeth Heggelund, Alasdair Mackenzie, Tobias Martinsen, Joel Benjamin Heim, Pavel Cheshev, Anna Bernardi, Ute Krengel

**Affiliations:** 1 Department of Chemistry, University of Oslo, P.O. Box 1033, NO-0315 Blindern, Norway; 2 Universita’ degli Studi di Milano, Dipartimento di Chimica, via Golgi 19, 20133 Milano, Italy; 3 School of Biomedical Sciences, University of Leeds, LS2 9JT Leeds, UK and School of Pharmacy, University of Oslo, P.O. Box 1068, NO-0316 Blindern, Norway; 4 Alere Technologies AS, Kjelsåsveien 161, NO-0884 Oslo, Norway; 5 Skolkovo innovation center, Office 229, OC Technopark bld. 2, Lugovaya str. 4, 143026 Moscow, Russia

## Abstract

Cholera is a life-threatening disease in many countries, and new drugs are clearly needed. *C*-glycosidic antagonists may serve such a purpose. Here we report atomic-resolution crystal structures of three such compounds in complexes with the cholera toxin. The structures give unprecedented atomic details of the molecular interactions and show how the inhibitors efficiently block the GM1 binding site. These molecules are well suited for development into low-cost prophylactic drugs, due to their relatively easy synthesis and their resistance to glycolytic enzymes. One of the compounds links two toxin B-pentamers in the crystal structure, which may yield improved inhibition through the formation of toxin aggregates. These structures can spark the improved design of GM1 mimics, either alone or as multivalent inhibitors connecting multiple GM1-binding sites. Future developments may further include compounds that link the primary and secondary binding sites. Serving as decoys, receptor mimics may lessen symptoms while avoiding the use of antibiotics.

## Introduction

The secreted enterotoxins from *Vibrio cholerae* and enterotoxigenic *E. coli* (ETEC) cause millions of diarrhoea episodes each year^1, 2^. Cholera is responsible for approximately 100,000 deaths annually, a number that has been predicted to increase with climate change^3^. ETEC mortality is estimated to be significantly higher, although it is difficult to determine accurate numbers due to underreporting and misdiagnosis^2^. With several epidemics in recent history, there is still a requirement for rapid-acting drugs. For example, the death toll of the 2010 Haiti cholera epidemic has reached over 9000; and after hurricane Matthew in October 2016^4^, the spectre of cholera looms again. Except for the newly licensed live-attenuated vaccine Vaxchora (PaxVax, U.S.A.), all current vaccines require two spaced doses, and are consequently not effective in an epidemic setting. No prophylactic drugs against cholera are currently on the market.

The cholera toxin (CT) and the heat-labile enterotoxin (LT) are AB_5_ toxins consisting of one catalytically active A-subunit bound to five non-toxic B-subunits arranged in a homopentamer^5^. The B-pentamers (CTB and LTB, respectively) are responsible for the binding to epithelial cells in the small intestine, facilitating the endocytosis of the toxin^6, 7^. Inside the intestinal cell, the A-subunit causes a signalling cascade leading to watery diarrhoea by the opening of ion channels. The resulting diarrhoea can be up to 1 litre/hour, leading to life-threatening dehydration if left untreated^8, 9^. Treatment is accomplished with the application of oral rehydration therapy, but this requires medical competence and large quantities of clean water, both of which can be limited resources during an epidemic. Antibiotics are also used in serious cases and have been shown to limit the duration of the disease by 50%^10^.

At present there are four cholera vaccines on the market; Shanchol (Shantha Biotechnics, India), Euvichol (EuBiologics, Korea), Vaxchora (PaxVax, USA) and Dukoral (Valneva, Sweden), the latter being effective towards both cholera and ETEC-induced diarrhoea^11^. Shanchol, Euvichol and Dukoral are inactivated vaccines that have to be taken in two spaced doses, and are hence impractical in a situation where rapid protection is required, such as during a cholera outbreak. They are most frequently used for travellers from non-endemic areas, and are not effective in children under the age of 1–2 years^12^. The only live attenuated vaccine, CVD 103-HgR, now licensed under the name Vaxchora, was recently approved for use by adults. A previous version of this vaccine (produced by Berna Biotech, formerly Swiss Serum and Vaccine Institute, Switzerland) was taken off the market in 2003 for financial reasons^13^. After re-assessment, it has been shown to be an effective vaccine that may be more suitable for use after outbreaks since it only requires one dose^14, 15^.

**Figure 1:**
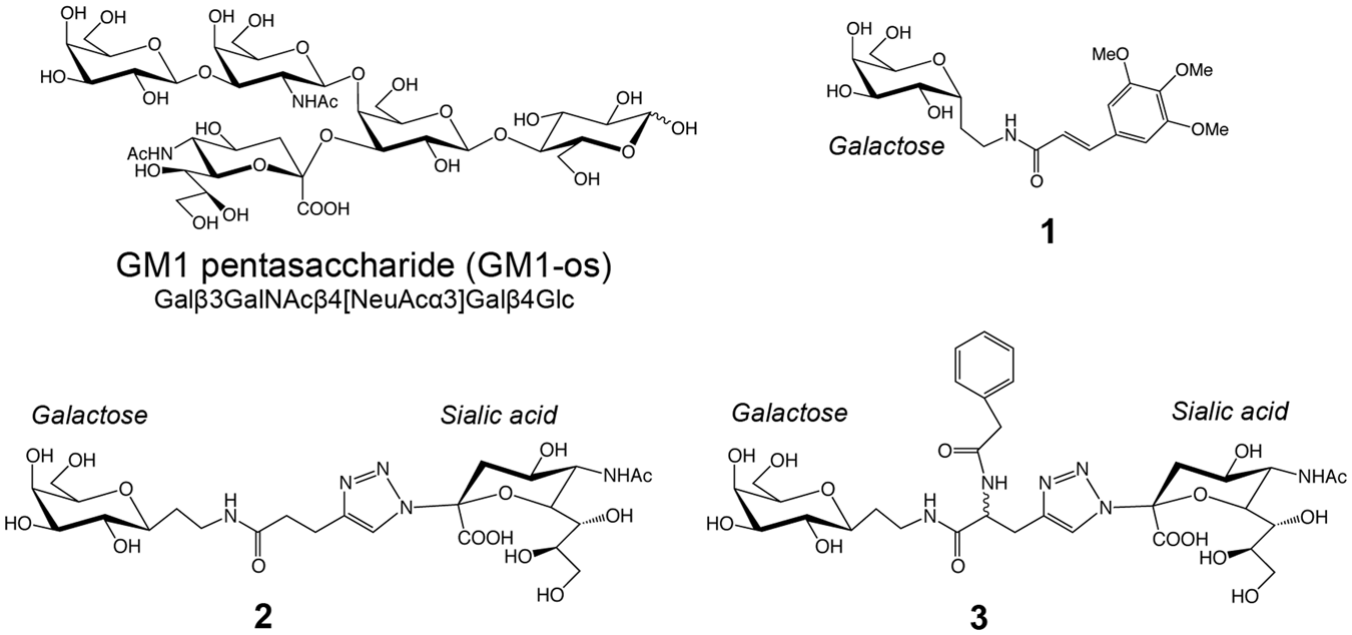
Schematic representation of GM1 pentasaccharide and inhibitors **1–3**. Carbohydrate residues are labelled, and the reducing end of GM1-os is indicated by a waved line. Compound **3** was used as a mixture of two stereoisomers (*R* and *S*), indicated by a waved line at the centre of the molecule. The figure was prepared with ChemBioDraw Ultra (PerkinElmer Informatics, Inc (www.cambridgesoft.com); version 12.0.3.1216).

The primary receptor of both CT and LT is the GM1 ganglioside^16^. The binding of CT to the GM1 oligosaccharide (GM1-os) Galβ3GalNAcβ4[NeuAcα3]Galβ4Glc (Fig. 1) is one of the strongest protein-carbohydrate interactions known, with a binding constant of 43 nM^17, 18^. Binding has been described as a “two-fingered grip”, provided by the two terminal residues, galactose (Gal) and sialic acid (NeuAc)^19, 20^. The specificity of this interaction is mainly determined by the terminal galactose residue, which is buried in a deep pocket at the rugged underside of the toxin (distant from the A-subunit). Methyl β-galactopyranoside (GalOMet) alone has been shown to bind to the CT with a *K*_D_ of 15 mM^18^. Binding of the sialic acid residue is not as strong (>200 mM), with its contribution resulting mainly from the conformational preorganization of GM1-os. CT and LT, with their five equivalent binding subunits, are known to be multivalent proteins. By binding five GM1 molecules simultaneously, the binding strength can be increased by at least an order of magnitude^21^.

In addition to GM1, the CT and related toxins have been shown to interact with fucosylated blood group antigens and derivatives^22–27^. Toxin binding to the blood group H-determinant Lewis-y (Fucα2Galβ4[Fucα3]GlcNAc) characteristic of blood group O, and its blood group A counterpart, have recently been characterized both crystallographically and by quantitative binding analysis^24^. The blood group antigens bind at the lateral side of the toxin, approximately 10 Å from the primary binding site. In all structures, the ligands are anchored to the toxin via a fucose residue. The binding affinity of the Lewis-y blood group determinant is in the millimolar range, and the ligand has been suggested to serve as a secondary receptor for cell entry^23, 24, 26, 27^. Indeed, recent cell biology experiments have shown that fucosylated carbohydrate structures can serve as functional receptors in cells in which GM1 synthesis is inhibited^28^. The cholera toxins come in two varieties: classical (c) and El Tor (ET). The two biotypes differ at two amino acid residues in the B-subunit, 18 and 47 (His18 and Thr47 in cCTB, and Tyr18 and Ile47 in ET CTB). While ET CTB displays reduced affinity for blood group A and B antigens^23, 24^, both CT variants bind equally strongly to GM1^29^. The classical CT was by far more abundant in ancient times, and is undergoing a resurgence again today^30^.

The search for an effective CT inhibitor was accelerated by the publications of the crystal structures of CT and LT in the 1990’s^19, 31^. The major strategy in inhibitor design has been to use the terminal galactose as an anchor, for example in the promising molecule *m*-nitrophenyl-α-d-galactopyranoside (MNPG). MNPG showed enhanced binding capability compared to d-galactose, presumably through the favourable displacement of a water molecule in the binding site, enabling the direct interaction of the nitro group of MNPG to Gly33^32^. Several crystal structures were solved to investigate the potential for MNPG and its derivatives as potent inhibitors^33–35^. Lately the search has turned towards multivalent inhibitors^36^, *e.g*. using pentavalent GM1-os on different scaffolds, creating a 1:1 interaction of toxin and inhibitor^37, 38^. An interesting approach is to “let CT fight itself” using CTB modified with GM1-os residues as penta-GM1-os-CTB neoglycoprotein inhibitors^39^. This potent glycoprotein inhibitor was shown to bind 1:1 to native CTB with picomolar affinity. Other potential CT inhibitors are polyphenolic compounds from grape-seed extract, suggesting that antagonists do not necessarily have to be based on carbohydrate structures^40, 41^.

**Figure 2:**
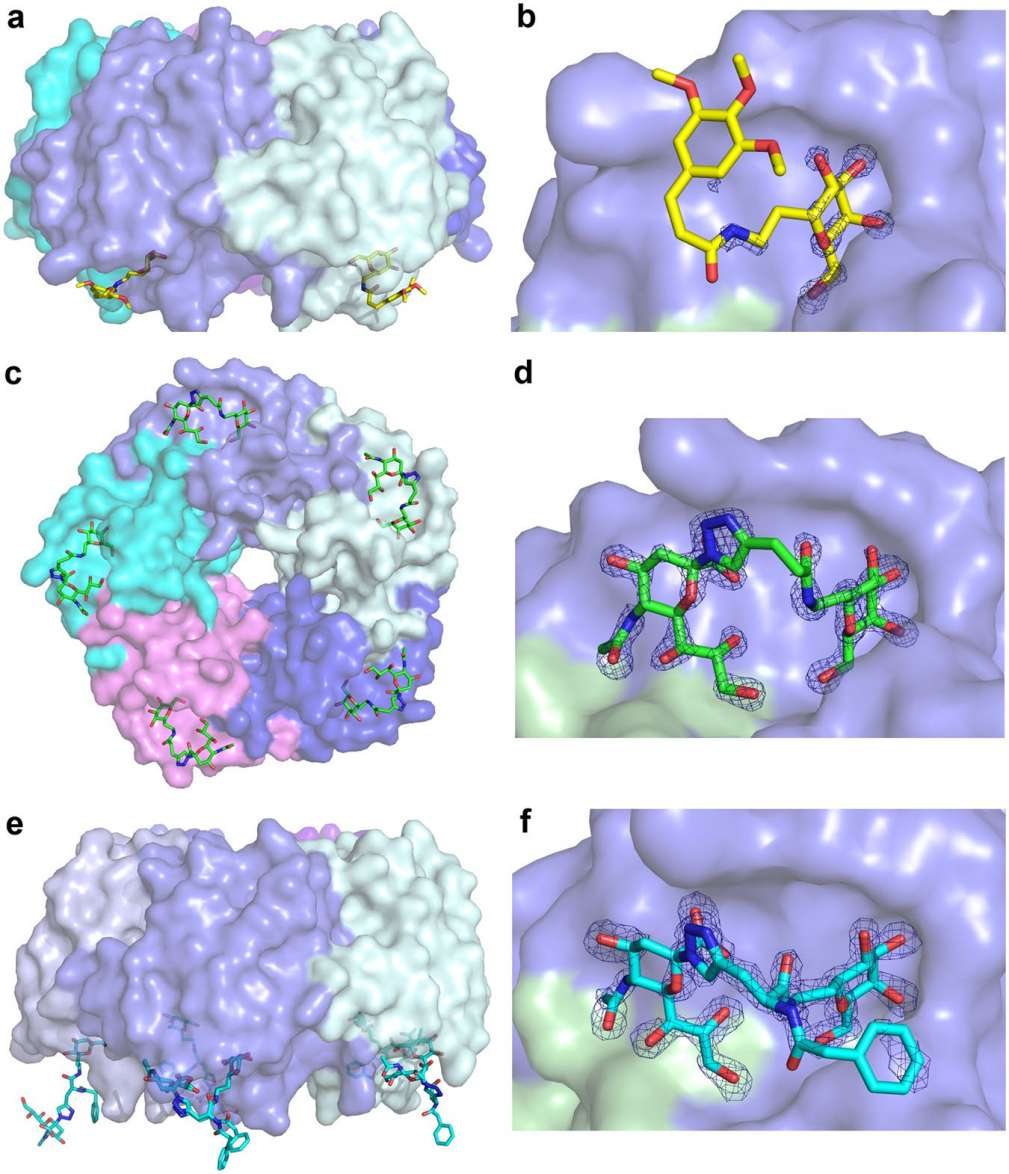
Structures of the toxin-antagonist complexes presented in this paper. The toxin B-pentamer is shown in 40% transparent surface representation, and the ligand in stick representation (**a**,**e**, side view; **b**,**c**,**d**,**f**, bottom view). Panels on the right show close-up views of the inhibitor binding sites, with σ_A_–weighted *F*_o_-*F*_c_ electron density maps contoured at 3.0 σ (generated before modelling the ligand). (**a**,**b**) ET CTB + **1** (yellow sticks), (**c,d**) ET CTB, in complex with **2** (green sticks), (**e,f**) pLTB R13H + **3** (cyan sticks). The figure was prepared with MacPyMol (Schrödinger LLC (www.schrodinger.com/pymol); version 1.8.0.2).

**Figure 3:**
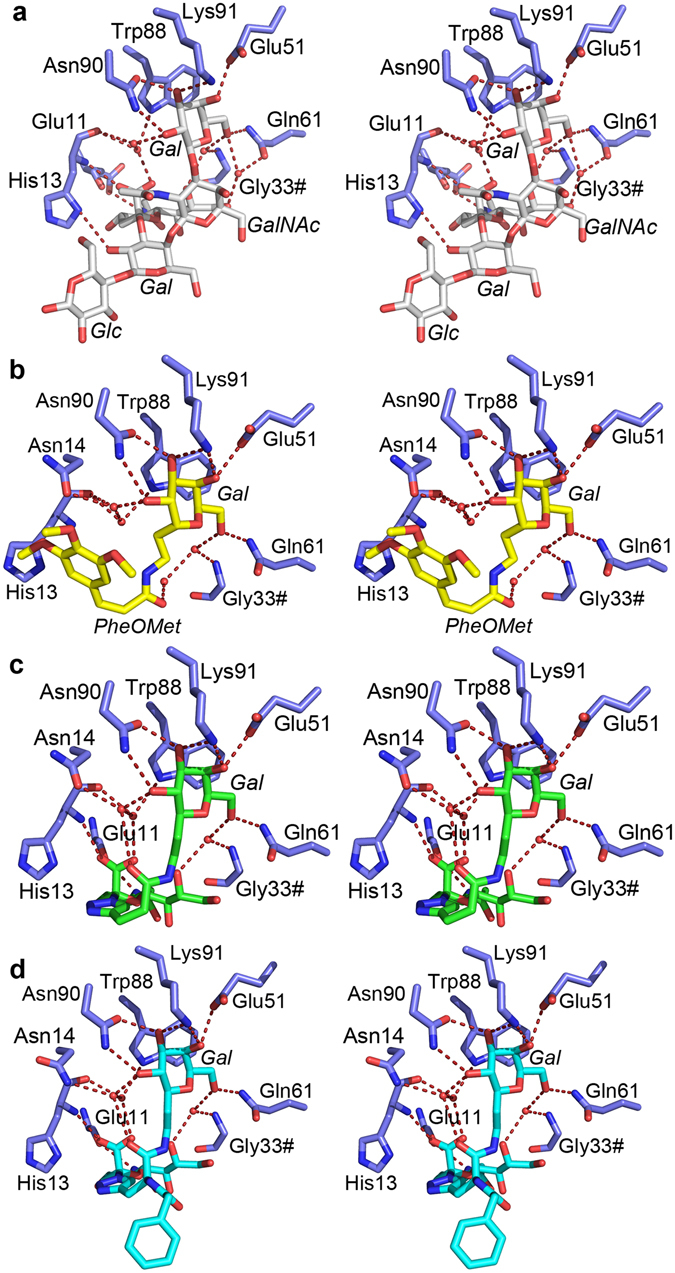
Stereoimages of the detailed carbohydrate-toxin interactions. Relevant amino acids are shown as blue sticks and labelled. Residues from neighbouring subunits are indicated with a hash (#). Water molecules are shown as red spheres, and hydrogen bonds by red dashed lines (restricted to bond lengths less than 3.6 Å, and with favourable angles). Carbohydrate residues are labelled in italics. Shown are toxin complexes with (**a**) GM1-os (white sticks; PDB ID 3CHB^20^), (**b**) **1** (yellow sticks), (**c**) **2** (green sticks), and (**d**) **3** (cyan sticks). All ligands bind to the same binding site, b-d show structures solved in this work. The figure was prepared with MacPyMol (Schrödinger LLC (www.schrodinger.com/pymol); version 1.8.0.2).

Despite the progress in chemo-enzymatic synthesis of GM1-os, the synthesis of this oligosaccharide is still expensive, and polyvalent GM1-os inhibitors are unlikely to result in a low-cost drug^42^. GM1 mimics have the potential to overcome this problem, and to result in materials that are relatively inexpensive and sufficiently active. Various approaches have been adopted to mimic GM1-os^43–45^. In particular, searching for non-hydrolyzable analogs, we explored both simple *C*-galactosides (Fig. 1, **1**)^46^ and bidentate ligands obtained by connecting a *C*-galactoside to a *N*-sialyltriazole residue (Fig. 1, **2** and **3**)^44^. These molecules are significantly simpler to synthesize than GM1-os and are stable to glycolytic enzymes, due to the absence of proper *O-*glycosidic linkages. In this paper, we describe the X-ray crystal structures of three such inhibitors (Fig. 1, **1**–**3**) binding to the cholera toxin and the heat-labile enterotoxin. Compound **1** ^46^ is a 3,4,5-trimethoxycinnamic acid galactoconjugate that can be synthesized without the need for protective groups, and has the potential for further extensions. Compounds **2** and **3** are bidentate ligands that combine the two terminal residues of the GM1 oligosaccharide, *i.e*. galactose and sialic acid^44^.

The binding strengths of all three ligands have been measured, and found to be in the upper μM range^44, 46^. An IC_50_ of 125 μM was measured for **1** in SPR competition experiments inhibiting CTB binding to immobilized asialofetuin^45^. The affinity of **2** and **3** for CTB was evaluated as 0.8–1.2 mM by weak affinity chromatography (WAC)^43^. For comparison, the affinity of the reference MNPG in the same experiment was 1.3 mM. While this is probably not enough for sufficient GM1 inhibition alone, the ligands have the potential for being combined into multivalent receptor-binding antagonists, linking several or all five binding sites.

## Results

We present four high-resolution structures of toxin-inhibitor complexes (Table 1; Fig. 2 and 3). To improve the chance of successful crystallization, we used three homologous toxins: cCTB, ET CTB and porcine LTB (pLTB; with the single-site substitution R13H). All three toxins have the same amino acid sequence in the primary binding site, and they have essentially identical 3D structures. pLTB forms crystals under a wider range of conditions than CTB, and the R13H mutation makes the primary binding site identical to that of CTB. Inhibitor **1** was crystallized with ET CTB, inhibitor **2** with cCTB and ET CTB, and inhibitor **3** with pLTB R13H. The cholera toxin structures presented here were solved to atomic resolution, allowing for detailed analysis of the interactions. They are only surpassed by the recently published cCTB structure in complex with Lewis-y, solved to 1.08 Å resolution (PDB ID: 5ELB)^24^. The pLTB structure is only surpassed by a structure solved to 1.3 Å (PDB ID: 1DJR)^33^, and is the first deposited structure of the R13H variant.

**Table 1:** Data collection and refinement statistics.

Data set	ET CTB + **1**	ET CTB + **2**	cCTB + **2**	pLTB + **3**
ESRF Beam line	ID29	ID23–1	ID23–1	ID14–1
Space group	*C*2	*C*2	*C*2	*P*2_1_2_1_2_1_
Unit cell parameters				
a	102.3	101.9	101.7	63.2
b	66.5	66.3	66.1	75.9
c	82.4	77.4	76.4	125.2
α	90	90	90	90
β	117.0	105.7	105.7	90
γ	90	90	90	90
Resolution (Å)	45.6–1.2 (1.27–1.20)	47.8–1.1 (1.19–1.12)	49.0–1.1 (1.19–1.13)	29.2–1.6 (1.63–1.60)
No. of unique reflections	144269 (17960)	139423 (4149)	135800 (4123)	74271 (2413)
Redundancy	2.9 (2.3)	2.7 (1.6)	2.8 (1.7)	2.9 (2.0)
Completeness (%)	93.8 (72.4)	74.3 (13.7)	73.7 (13.9)	92.9 (61.7)
*I*/*σ* (*I*)	5.6 (0.7)	8.9 (0.7)	6.9 (0.7)	10.9 (3.1)
*R*_meas_	12.1 (>100)	6.5 (>100)	8.4 (96.7)	7.2 (30.6)
CC_1/2_	99.4 (33.7)	99.9 (35.9)	99.7 (50.4)	99.6 (90.2)
*R*_cryst_/*R*_free_ (%)	19.5/22.8	17.5/19.8	19.7/22.9	14.7/17.6
r.m.s.d bond length (Å)	0.021	0.019	0.028	0.022
r.m.s.d bond angles (°)	2.15	1.90	2.50	2.24
Average *B*-factor (Å^2^)				
Backbone	15.1	16.2	16.1	10.4
Side chains	18.1	17.7	17.8	12.9
Water	25.0	25.2	26.3	24.9
Inhibitor	35.6	19.4	19.2	15.9
All atoms	18.0	18.2	18.3	14.1
Ramachandran plot profile (%)				
Favoured	97.7	97.3	97.0	97.6
Allowed	2.3	2.7	3.0	2.4
Disallowed	0.0	0.0	0.0	0.0
PDB ID	5LZJ	5LZG	5LZH	5LZI

Values in parentheses refer to the highest resolution shells. *R*_cryst_ = Σ||*F*_o_|−|*F*_c_||/Σ|*F*_o_|, where |*F*_o_| and |*F*_c_| are the observed and calculated structure factor amplitudes, respectively.

### Crystal structure of ET CTB in complex with inhibitor 1

The toxin complex with inhibitor **1** crystallized in space group *C*2, and the structure was refined to 1.2 Å (Table 1). Sufficient electron density for **1** was present in two out of five binding sites (chains C and D); two sites were occluded by crystal contacts, and the remaining site had too weak density to model the ligand with confidence. **1** binds with its galactose moiety in the galactose-binding pocket, interacting with Glu51, Gln61, Asn90 and Lys91 (Figs 2a,b and 3b). The aryl moiety seems to be disordered in the crystal structure and is not clearly bound in the hydrophobic patch, differing from previous predictions^47^. No clear electron density is visible for the ligand after the peptide bond (Fig. 2b). The binding sites lacking bound ligand display less defined loop regions (residues 50–60; chains A, B and E), indicating that ligand binding causes ordering of the CTB structure.

### Crystal structures of cCTB and ET CTB in complex with inhibitor 2

Both toxin complexes with inhibitor **2** crystallized in space group *C*2, exhibiting the same crystal form as ET CTB co-crystallized with **1**. The crystal structures were solved to 1.1 Å resolution and contain one B-pentamer in the asymmetric unit. Both CT variants have well-defined electron density for inhibitor **2** in all binding sites (Fig. 2d). The five crystallographically independent binding sites show unprecedented details of the inhibitor-protein interactions. As in the other structures, the galactose moiety of **2** is buried in the GM1 binding site, H-bonding with Glu51, Gln61, Asn90 and Lys91 (Figs 2c,d and 3c). The sialic acid residue has direct interactions with the backbones of Glu11 and His13. The differences to GM1-os binding can be seen in the linker region of **2**, where the hydrogen bond of the core Gal to the side chain of His13 is no longer achievable. Instead, closer to the terminal Gal, the carbonyl group of the linker engages in a water-mediated interaction to Asn14 (Fig. 3a,c). The ligand binding mode was predicted by earlier modelling experiments^44, 47^.

### Crystal structure of pLTB R13H in complex with inhibitor 3

**Figure 4:**
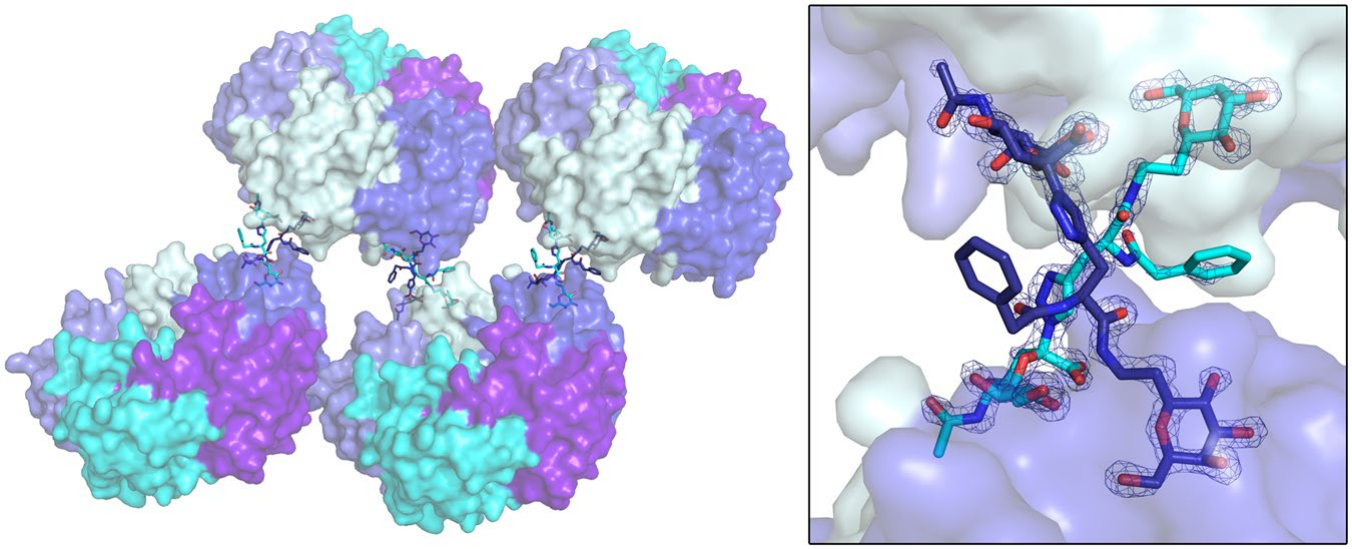
Cross-linking of toxin B-pentamers by **3** in the crystal structure. The toxins are shown in surface representation, with the inhibitor in cyan and dark blue sticks. The inset shows a close-up view of the binding interaction, with two antagonists (cyan and dark blue sticks) bridging two adjacent toxins. The σ_A_–weighted *F*_o_-*F*_c_ electron density map is shown for both inhibitors (contoured at 3.0 σ, generated before modelling the ligand). The figure was prepared with MacPyMol (Schrödinger LLC (www.schrodinger.com/pymol); version 1.8.0.2).

The toxin complex with inhibitor **3** was solved to a resolution of 1.6 Å in space group *P*2_1_2_1_2_1_ with one B-pentamer in the asymmetric unit. The inhibitor was present in all five binding sites (Fig. 2e,f), and binds in the same manner as **2**, with the galactose residue binding to Glu51, Gln61, Asn90 and Lys91, and the sialic acid residue interacting with Glu11 and His13 (Fig. 3d). The benzylamido extension, the feature distinguishing this inhibitor from **2**, is flexible and sometimes adopts different conformations. Interestingly, in two of the binding sites, the inhibitor is stretched out and links two adjacent toxin pentamers (Fig. 4). In these two cases, the sialic acid moiety extends into the sialic acid binding site of another B-pentamer, creating a cross-over with another ligand. The inhibitor solution was a mixture of two stereoisomers, as indicated by the waved line in Fig. 1. Both isomers displayed very similar retention factors on weak affinity chromatography with immobilized CTB^48^, suggesting a similar affinity for the protein^44^. The *S*-isomer is predominantly seen in the crystal structure, such that only one of the five binding sites contains the *R*-isomer.

### Cross-linking with inhibitor 3 also occurs in solution

To test whether inhibitor **3** also induces cross-linking in solution, we collected SAXS data for ligand-free and ligand-bound pLTB R13H. The major fraction of ligand-free and ligand-bound toxin was found as free B-pentamer (64–100%, Supplementary Fig. S1). A small concentration-dependent increase in oligomerization can be observed for ligand-free toxin. Upon the addition of a 10-fold molar excess of inhibitor **3**, the *R*_g_ and *I*(0) values increased for all four concentrations tested, corresponding to an increase in cross-linked CTB-dimers by approximately 20% (Supplementary Fig. S1). Hence, our data show that the presence of inhibitor **3** shifted the equilibrium towards CTB-dimers (“decamers”) (Supplementary Fig. S1).

## Discussion

Here we present the X-ray crystal structures of four enterotoxin inhibitor complexes at atomic resolution. As expected, all three inhibitors bind in the primary binding site, acting as decoys for the toxins’ main receptor, the GM1 ganglioside. By using these simple *C*-glycosidic mimics of GM1-os, it might be possible to simplify the organic synthesis process, which is a crucial step in the development of a low cost drug.

The inhibitors’ binding affinities are in the upper micromolar range, and are unlikely to result in potent inhibitors without modification. The combination of simple inhibitors into multivalent constructs has been proposed before as effective antagonists of CT action in *Vibrio cholerae* infections^42, 49^. One of the inhibitors shows the surprising capability to facilitate bridging to nearby toxins. Ligand-induced dimerization of toxin B-pentamers has been reported previously, both for the CTB^50^ and the shiga-like toxin^51^. More recently, Turnbull, Zuilhof and co-workers^52^ found that divalent and tetravalent analogues of GM1 were better inhibitors than pentavalent inhibitors^53, 54^. Likewise, earlier reports found a 47,500-fold increase in binding for octavalent GM1-os dendritic glycoconjugates, resulting in an IC_50_ of 5 ± 1 × 10^−11^ M^55^. This is conceivably achieved through linking more than two B-pentamers together, resulting in the formation of aggregates. The bivalent inhibitor described in this paper connects receptor binding sites from different pentamers in the crystal unit cell, creating a chain of toxins (Fig. 4). This would have been difficult to predict by molecular modelling, which only deals with one B-pentamer at a time. The small increase in oligomerization in solution (20%) measured with SAXS is likely not sufficient to be medically relevant, and the stronger cross-linking effect *in cristallo* was probably selected for by crystallization. Nevertheless, it could represent a valuable starting point for designing cost-effective multivalent constructs designed to cross-link the toxins in solution. Aggregating the soluble toxin could be a very effective strategy for preventing fluid accumulation during cholera infection.

By exploiting the blood-group antigen binding site of the toxin, it might be possible to create even more potent inhibitors that function by promotion of the aggregation effect. It was recently shown that the two binding sites for GM1-os and blood group antigens can be occupied simultaneously^22, 24^. Dual-binding site inhibitors could have the potential to induce an aggregation event by linking the primary site from one B-pentamer to the secondary site of another. Although B-pentamers in a crystal are likely positioned closer together than in the gut of a cholera-infected individual, inhibitor-induced linking of pentamers in the human gut is conceivable. The concentration of CT in human stool has been measured at 10 μg/ml^56^, and it is likely that the local concentration in the small intestine, at the sites where the toxins are secreted by the bacteria, is significantly higher. Indeed, other reported inhibitors may work by linking different binding sites in different B-pentamers, for example the high-molecular weight polysaccharide from garlic water extract that has been shown to be bioactive against CTB^57^.

The structural insights offered in this paper can help to spark further developments in the design of potent and cost-effective cholera toxin inhibitors, especially by exploiting the promising tactic of dual-site binders and multivalent compounds. The development of such inhibitors will be crucial for making a prophylactic cure against cholera and ETEC-induced diarrhoea, while at the same time avoiding antibiotics. More than 1.8 billion people use a drinking-water source contaminated with faecal matter, and in addition local conflicts and natural disasters can trigger epidemics^58^. The development of a low-cost drug that is independent of a cold-chain delivery will have a great potential to lessen the incredible toll these diseases have on developing nations.

## Materials and Methods

### Synthesis of GM1 mimics 1–3

Inhibitor **1** ^46^ uses one component of the binding epitope, galactose, mounted on a cinnamic acid-based spacer. Inhibitor **2** ^44^ mimics the GM1 oligosaccharide by connecting a sialic acid residue and galactose with a spacer consisting of a triazole group and an amide bond. Inhibitor **3** is identical to **2** except for an added benzylamido moiety.

The synthesis of **1** (compound 11) is described in^46^, and the syntheses of **2** (compound 44) and **3** (compound 57) are described in^44^. In short, **1** was synthesized starting from easily accessible α-*C*-allyl galactoside^59^ that was transformed in three steps into 2-(α-d-galactopyranosyl)ethylamine, which was finally acylated with the appropriate cinnamoyl chloride. The same starting material (α-*C*-allyl galactoside) was used for the synthesis of **2** and **3** through 2-(α-d-galactopyranosyl)acetaldehyde. The latter was isomerized using the Massi-Dondoni protocol^60^ to the β-*C*-glycoside, which was transformed into 2-(β-d-galactopyranosyl)ethylamine in two steps. Elongation with 4-pentynoic acid, followed by a click reaction with sialyl azide^61^ under Sharpless conditions yielded **2**. The branched derivative **3** was obtained as **2**, by replacing pentynoic acid with commercially available racemic propargyl glycine.

### Expression of ET CTB

The gene for ET CTB (Uniprot: P01556) was previously introduced into the non-pathogenic *Vibrio* sp. 60 under an IPTG-inducible promoter^62^, an expression system kindly provided by Professor Timothy Hirst. The gene includes an N-terminal signal sequence directing the protein to the periplasmic space and subsequent secretion into the growth media, facilitating high expression and easy purification. The bacteria were grown in high-salt LB medium (15 g/L NaCl) supplemented with 0.1 mg/ml ampicillin at 30 °C with shaking. Expression was induced with 0.5 mM IPTG at an OD_600nm_ of 0.2, followed by protein production for 16–20 hours. The medium was separated from the bacterial pellet by centrifugation at 40,000 *g* at 20 °C, and purified further as described for all toxin B-pentamers.

### Expression of cCTB and pLTB R13H

The genes for cCTB (Uniprot: Q57193) and pLTB R13H (Uniprot: P32890 with an Arg to His substitution) were previously synthesized, cloned into the pET21b + plasmid, and introduced into *E. coli* BL21(DE3)^24, 63^. The sequence contains the signal sequence for secretion, but the protein is retained within the periplasmic space of *E. coli*, requiring purification by periplasmic lysis. The bacteria were grown in LB medium containing 0.1 mg/ml ampicillin at 37 °C until an OD_600nm_ of 0.5 was reached. After the temperature was lowered to 25 °C, expression was induced with 0.5 mM IPTG, and protein produced for 14–18 hours. The supernatant was separated from the bacterial pellet by centrifugation at 6900 *g* and the pellet was re-suspended in ice-cold periplasmic lysis buffer (30 mM Tris pH 8, 20% (w/v) sucrose, 1 mM EDTA, 5 mM MgSO_4_), with 150 μg lysozyme per gram of cell pellet added after re-suspension. The periplasmic solution was kept cold while stirring for 10–30 minutes. The solution was centrifuged at 8500 *g* for 15 minutes and dialyzed against PBS in a Snakeskin tube (Thermo Scientific, 3500 MWCO). The resulting protein was centrifuged at 45,000 *g* for 20 minutes and purified further as described for all toxin B-pentamers.

### Purification of toxin B-pentamers

The protein was applied to a d-galactose-sepharose affinity column (Thermo Scientific) and eluted using 300 mM galactose in PBS. The fractions were concentrated using Vivaspin 20 ml concentrator tubes (5000 MWCO, PES membrane, Sartorius), and subjected to size-exclusion chromatography on a Superdex75 column mounted on an Äkta FPLC machine, pre-equilibrated with a Tris running buffer (20 mM Tris, 200 mM NaCl at pH 7.5). Fractions with toxin were dialyzed against the Tris running buffer in a Snakeskin dialysis tube (3500 MWCO, Thermo Scientific), concentrated using concentrator tubes to 3–10 mg/ml, and stored at −80 °C.

### Co-crystallization of toxins with inhibitors

Two hours before crystallization, toxins and inhibitors were mixed at a molar ratio of 1:10 (B-subunit:inhibitor). Initial co-crystallization experiments were carried out at 20 °C with a crystallization robot (Oryx4, Douglas Instruments). First hits were obtained in both the Morpheus screen^64^, in conditions A1, A4, A9, A10 and A12, and the PGA-LM screen conditions D10 and D5 (both Molecular Dimensions). The hits were optimized using the hanging-drop vapour-diffusion technique using 24-well trays. Variations in the conditions identified by preliminary screening were subsequently explored with the use of microseeding, where seeds were prepared by crushing crystals from earlier screens with a seed bead. pLTB R13H was chosen for co-crystallization with inhibitor **3** due to its relative ease of crystallization.

The ET CTB + **1** data set was collected from a crystal found in an almost dried-out drop containing MES pH 6, 30% PEG 400, 3% PGA-LM (optimization of PGA-LM condition D5). The drop was hydrated with the same buffer, and no additional cryo-protection was necessary.

Diffraction-quality crystals were obtained for ET CTB + **2** in 0.1 M MES/Imidazole buffer, pH 6.5, with 10% PEG 1000, 10% PEG 3350, 10% MPD and 0.03 M divalent cations (optimization of the Morpheus A4 condition). cCTB + **2** crystals were obtained in 0.1 M MES/Imidazole buffer pH 6.5, with 8% PEG1000, 8% PEG 3350, 8% MPD and 0.03 M divalent cations. These crystals were cryo-protected using a buffer containing 0.1 M MES/Imidazole buffer, pH 6.5, with 12.5% PEG 1000, 12.5% PEG 3350, 12.5% MPD and 0.03 M divalent cations.

Crystals of the pLTB R13H + **3** complex were obtained from several conditions of the Clear Strategy 1 crystal screen (Molecular dimensions). Diffraction-quality crystals were grown in 0.1 M sodium cacodylate pH 6.5, 0.2 M lithium sulphate, and 15% PEG 4000, and cryo-protected using the original buffer with 25% glycerol added.

### Data collection and refinement

Samples were mounted in loops, flash-frozen in a nitrogen cryo-stream, and subjected to data collection at the ESRF, Grenoble, France^65–67^ (Table 1). Scaling and processing of the CTB data sets was done with XDS^68^, whereas the pLTB data set was scaled and processed with *Mosflm* ^69^. Diffraction cut-offs were chosen based on the assessment of CC_1/2_^70, 71^ (Table 1), and the quality of the electron density. The pLTB + **3** diffraction data were collected using remote access, to a resolution of 1.60 Å, which in hindsight turned out to be a very conservative cut-off with CC_1/2_ = 90.2. The structures were solved by molecular replacement using *MOLREP* ^72^ from the *CCP4* software suite^73^. The search model used for the CTB structures was a 1.25 Å crystal structure of cCTB (PDB ID: 3CHB^20^). The pLTB R13H structure was solved using the native pLTB crystal structure (PDB ID: 1EFI^74^) as a search model. The search models were prepared by pruning unconserved residues and removing water molecules with the program *CHAINSAW* ^75^. The inhibitors were modelled in MarvinSketch and MarvinSpace (ChemAxon.com), and the corresponding PDB- and library files were created using *PRODRG* ^76^. After initial rigid body refinement using *REFMAC5* ^77^, setting 5% of the reflections aside for calculating the *R*_free_, the structures were surveyed and patched with *Coot* ^78^ and further refined. At later stages of the refinement, water molecules were manually added with Coot. The inhibitors were included last. All inhibitors were modelled with 100% occupancy after assessment of the difference electron density and *B*-factors of the ligand and the nearby protein chain.

The data sets with inhibitor **2** showed significant anisotropy, which resulted in disproportionally high *R*-factors, a common effect of anisotropy. Although the Hamilton *R* ratio test showed that the structures could also be refined with anisotropic *B*-factors, this was abandoned in favour of an isotropic *B*-factor model with TLS refinement, since anisotropic *B*-factors resulted in inferior electron density in the loop areas (residues 50–60), and made model building harder. The application of TLS refinement resulted in well-defined electron density also in the loop areas, along with *R*/*R*_free_ values comparable to those obtained from anisotropic *B*-factor refinement. In the structure of cCTB in complex with **2** some of the intramolecular disulphide bridges (Cys9-Cys86) show oxidation, suggesting radiation damage. Cys9 has previously been reported to have two conformations, one of them pointing away from Cys86 and Thr15^20, 24^. However, in this structure, strong positive difference density (10 r.m.s.d.) was observed on the opposite site of the disulphide link, towards Thr15, indicating a modification of the sulphur atom rather than an alternative conformation of the residue. Partial oxidation of the residues was modelled by replacing the cysteines with S-oxy cysteine (CSX), with 50% occupancy of the oxygen in chains A, B and D.

The pLTB R13H + **3** data set is of high quality with close to 100% completeness, while the ET CTB + **1** data set exhibited some anisotropy. Both structures were refined with standard isotropic *B*-factors.

### Small-angle X-ray scattering

SAXS data were collected at the automated BM29 bioSAXS beamline at the ESRF, Grenoble, France with a PILATUS 1 M detector (Dectris)^79^. The data were obtained using an energy of 12.5 keV, a beam size of 700 × 700 μm and at a detector distance of 2.87 m covering a *q*-range of approximately 0.0365 nm^−1^ < *q* < 4.93 nm^−1^ (*q* = 4π sin (θ/2)/λ, where λ is the wavelength and θ the scattering angle) after calibration with water and bovine serum albumin. All measurements were carried out at 20 °C in Tris buffer (20 mM Tris, 200 mM NaCl at pH 7.5). Protein stock solution at 3.1 mg/ml was either mixed with water or with inhibitor **3** at a molar ratio of 1:10 (B-subunit:inhibitor). Samples of lower protein concentration were obtained by successive 1:2 dilutions with buffer, resulting in a concentration range of 0.39–3.1 mg/ml. The 45-μL samples were run through a capillary using the flow mode of the automated sample changer^80^. SAXS data were collected in ten successive frames of 2 s each to monitor radiation damage. Data reduction was done using the standard tools at BM29^81, 82^. The *R*_g_ (radius of gyration) and forward scattering intensity *I*(0) at zero angle values were estimated from the scattering data using Guinier analysis. Fractions of B-pentamers and decamers were calculated with the assumption that *M*_Pentamer_ corresponds to *I*(0) of ligand-free toxin at the lowest protein concentration (*I*(0) = f_Pentamer_ *M*_Pentamer_ + f_Decamer_(2*M*_Decamer_)).

## Electronic supplementary material

Supplementary Information
