# Supplementary material for: Towards new cholera prophylactics and treatment: Crystal structures of bacterial enterotoxins in complex with GM1 mimics
Source: Sci Rep. 2017 May 24;7:2326. doi: 10.1038/s41598-017-02179-0 (PMC5443773; doi:10.1038/s41598-017-02179-0)
Supplement: Supplementary file 1 — Supplementary Information [file 41598_2017_2179_MOESM1_ESM.pdf]

SUPPLEMENTARY MATERIAL

Towards new cholera prophylactics and treatment: Crystal structures of bacterial enterotoxins in complex with GM1 mimics

Julie Elisabeth Heggelund[1,3], Alasdair Mackenzie[1,3], Tobias Martinsen[1], Joel Benjamin Heim[1], Pavel Cheshev[2], Anna Bernardi[2], Ute Krengel[1*]

[1]Department of Chemistry, University of Oslo, P.O. Box 1033, NO-0315 Blindern, Norway

[2] Universita' degli Studi di Milano, Dipartimento di Chimica, via Golgi 19, 20133 Milano, Italy

[3]Present addresses: J.E. Heggelund, School of Biomedical Sciences, University of Leeds, LS2 9JT Leeds, UK and School of Pharmacy, University of Oslo, P.O. Box 1068, NO-0316 Blindern, Norway; A. Mackenzie, Alere Technologies AS, Kjelsåsveien 161, NO-0884 Oslo, Norway; P. Cheshev, Skolkovo innovation center, Office 229, OC Technopark bld. 2, Lugovaya str. 4,143026, Moscow.

*Correspondence should be addressed to J.E.H. (j.e.heggelund@farmasi.uio.no) or U.K. (ute.krengel@kjemi.uio.no).

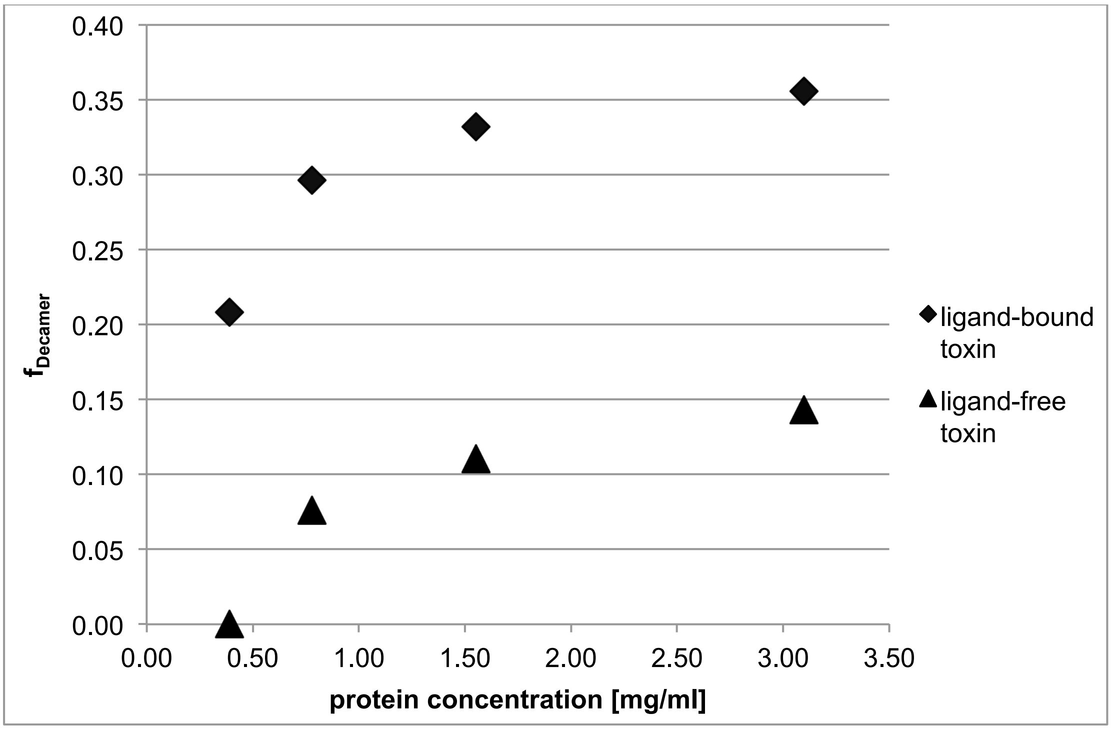

Supplementary Figure 1. Analysis of toxin oligomerization in solution by SAXS measurements on pLTB R13H.

Ligand-free toxin and toxin bound to compound **3** were exposed to X-rays in solution. The observed forward scattering intensity, which is proportional to the molecularity, was transformed into pentamer and decamer fractions. The decamer fraction is plotted against the protein concentration. Ligand-free toxin shows a small concentration dependent increase (0-15%). Upon the addition of a 10-fold molar excess of inhibitor **3**, the R_g and $I(0)$ values increased for all four concentrations tested, corresponding to an increase in decamer formation (or cross-linked CTB-dimers) by approximately 20%.
